# Phage-Derived Antibacterials: Harnessing the Simplicity, Plasticity, and Diversity of Phages

**DOI:** 10.3390/v11030268

**Published:** 2019-03-18

**Authors:** Bi-o Kim, Eun Sook Kim, Yeon-Ji Yoo, Hee-Won Bae, In-Young Chung, You-Hee Cho

**Affiliations:** Department of Pharmacy, College of Pharmacy and Institute of Pharmaceutical Sciences, CHA University, Gyeonggi-do 13488, Korea; ayumm82@gmail.com (B.-o.K.); eskim@cha.ac.kr (E.S.K.); roouge@naver.com (Y.-J.Y.); whitebb0412@cha.ac.kr (H.-W.B.); iychung@cha.ac.kr (I.-Y.C.)

**Keywords:** phage, engineering, lysin, pyocin, life cycle

## Abstract

Despite the successful use of antibacterials, the emergence of multidrug-resistant bacteria has become a serious threat to global healthcare. In this era of antibacterial crisis, bacteriophages (phages) are being explored as an antibacterial treatment option since they possess a number of advantages over conventional antibacterials, especially in terms of specificity and biosafety; phages specifically lyse target bacteria while not affecting normal and/or beneficial bacteria and display little or no toxicity in that they are mainly composed of proteins and nucleic acids, which consequently significantly reduces the time and cost involved in antibacterial development. However, these benefits also create potential issues regarding antibacterial spectra and host immunity; the antibacterial spectra being very narrow when compared to those of chemicals, with the phage materials making it possible to trigger host immune responses, which ultimately disarm antibacterial efficacy upon successive treatments. In addition, phages play a major role in horizontal gene transfer between bacterial populations, which poses serious concerns for the potential of disastrous consequences regarding antibiotic resistance. Fortunately, however, recent advancements in synthetic biology tools and the speedy development of phage genome resources have allowed for research on methods to circumvent the potentially disadvantageous aspects of phages. These novel developments empower research which goes far beyond traditional phage therapy approaches, opening up a new chapter for phage applications with new antibacterial platforms. Herein, we not only highlight the most recent synthetic phage engineering and phage product engineering studies, but also discuss a new proof-of-concept for phage-inspired antibacterial design based on the studies undertaken by our group.

## 1. Introduction

Since the commercialization of penicillins in the 1940s, antibiotics have been used worldwide not only as medicines for human populations, but also as additives for feed in the livestock industry. Due to this misuse and overuse of antibiotics, life-threatening superbugs or multidrug-resistant (MDR) bacteria have been, primarily through genetic evolution, rapidly emerging to become one of the greatest threats to humankind [1,2]; in Europe, about 400,000 people were infected by MDR bacteria in 2007, causing €1.5 billion worth of losses in hospital costs and economic productivity [3]. As for deaths from MDR infections in the United States alone, approximately 23,000 people die annually from infections involving MDR bacteria and unless appropriate antibiotic treatments are provided, the estimated number of global deaths annually will rise to 10 million by 2050 [4,5,6]. Although there is an urgent need for new antibacterials to overcome these MDR bacterial infections, because the development of drugs for chronic diseases which can be used widely without loss of efficacy is more lucrative, interest in antibiotics by the pharmaceutical industry has significantly dwindled.

However, since the worldwide antibacterial crisis is not only an issue of money or human welfare, but one of human survival, many countries have actively engaged in assisting or incentivizing pharmaceutical companies back into antibacterial research and development. For example, in the USA, the Generating Antibiotic Incentives Now (GAIN) Act was signed into law in 2012, which extends drug-patent exclusivity for an additional five years and fast-tracks the FDA-approval process. The EU-funded Innovative Medicine Initiative (IMI) kicked off the New Drugs for Bad Bugs (ND4BB) program in 2013. The WHO started promoting the Global Antimicrobial Resistance Surveillance System (GLASS) program in 2015 and announced the prioritization of antibiotic development, encouraging member states to collectively achieve the program’s goals.

In spite of these endeavors, many scientists are concerned about the very nature of antibiotics; the more they are used now, the less effective they become later. Hence, spurred on by recent advancements in scientific knowledge, there have been calls for the exploration of new-paradigm, or first-in-class, antibacterial drugs to mitigate the emergence of drug resistance, so that humans can stay ahead of bacteria in the everlasting war with them. One of the approaches in this regard has been to revive the exploration of old natural antibiotics, bacteriophages (phages). Phages are biological entities, which are continuously coevolving alongside their host bacteria even as the latter evolve so as to survive with their human or animal hosts [7]. Furthermore, phages are “precision” antibacterials that specifically kill only the target bacterial species or strains and have little effect on commensal microflora [8,9]. They also make for “self-replicating” or “intelligent” drugs, in that their antibacterial activity is amplified (via phage multiplication) and localized at the site of infection and in that they are effective only until the target bacteria disappear. Another advantage is that phages are biologics with little known toxicity or side effects, which ensures their cost-effective development and production [10]. After their discovery in the early 20th century, phages were harnessed to control bacterial infections in Eastern Europe and the former Soviet Union. Much attention has been paid recently to phages in Western countries, as exemplified by the EU-funded Phagoburn study for burn patients infected with *Escherichia coli* and *Pseudomonas aeruginosa* [11]. In addition to clinical trials for human diseases, phages have also been used to control crop diseases [12,13,14,15] and to ensure food safety [16,17].

Despite the advantages of phages over conventional antibiotics, they have some disadvantages as well, which inevitably stem from the fact that phages are relatively large in size and highly specific to bacterial strains. The overall particle size of phages ranges from about 20–200 nm, with some larger phages being rapidly recognized by the reticuloendothelial system, resulting in the subsequent loss of effective phages. Phage materials are immunogenic and trigger immune responses upon entrance to the blood stream [16,17,18,19]. The high specificity is sometimes disadvantageous given that only a set of particular strains are targeted by the phages [9]. Phages are able to horizontally transfer (transduce) genetic material between host bacteria, which drives the evolution of the bacterial population in such a way that they can enhance the fitness of the bacteria. Especially concerning is the possibility of transfer of the genes coding for virulence and/or antibiotic resistance. More importantly, Modi et al. [20] showed that antibiotic treatment leads to the broad enrichment of phage-encoded genes involved in antibiotic resistance, indicating the role of the phageome in the emergence and spread of MDR bacteria during antibiotic treatment [20].

Clearly, the exploration of natural habitats for more diverse phage reservoirs is necessary to rapidly expand the available library of phage resources. In the meantime, further scientific research is required to maximize the utility of current phage resources by minimizing, or obviating, the disadvantageous aspects of phages. Recently advanced recombineering-based genetic engineering tools and next-generation sequencing (NGS) technology are applicable to the Earth’s largest genetic resource.

In this review, the utilities of phages and phage proteins are discussed and the major approaches for phage applications summarized in three sections: (1) The mixing and matching of phages and/or other genetic resources to create new phages or phage-like particles (synthetic phage engineering); (2) the utilization and/or engineering of phage-derived proteins to create new antibacterial proteins (phage protein engineering); and (3) the utilization of phage proteins to provide new antibacterial platforms with known molecular mechanisms (phage-inspired antibacterial design).

## 2. Synthetic Phage Engineering

**Figure 1:**
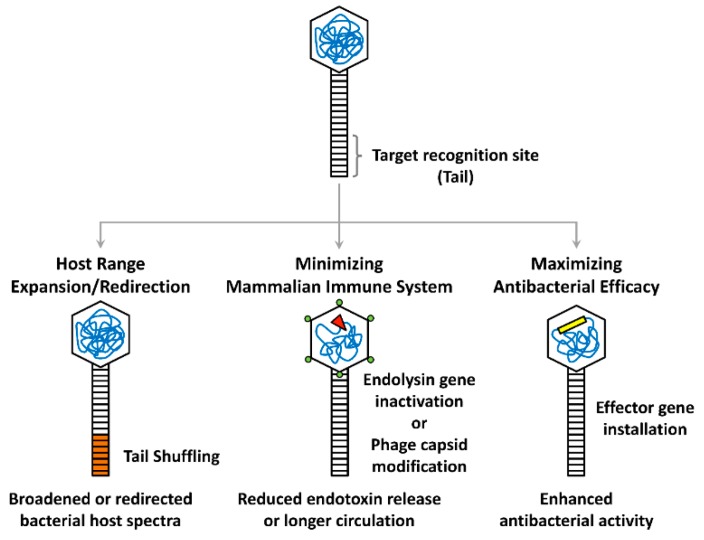
Synthetic phage engineering strategies. Major strategies for modifying the target recognition site (tail), phage head (hexagonal), or genome (tangled lines) are depicted. The bacterial host spectrum of a phage can be expanded or redirected by tail shuffling (orange), while mammalian host immune responses can be reduced or minimized by insertional inactivation of the endolysin gene (red triangle) or capsid modification (green circles). Heterologous effector genes (yellow rectangle) can also be installed on the phage genome to maximize the antibacterial efficacy.

Recent advancements in synthetic biology tools have enabled the development of novel phage genome engineering methods to create specialized or designer phages. These synthetic phages are installed with newly amended or improved functionalities, as depicted in Figure 1. Despite the wide range of biotechnological applications for synthetic phages, which also include those for protein/peptide display and drug delivery, only antibacterial phages are focused on here. In this section, current phage engineering approaches are described and classified into three strategies, two of which are aimed at overcoming the current limitations of phage therapies (such as narrow specificity and host immunity), and the third of which targets the enhancement of native antibacterial properties in a variety of ways.

### 2.1. Phage Engineering to Broaden or Redirect Antibacterial Spectra

Phages are natural antibacterial agents that target specific bacteria without affecting others. However, any individual phage typically has its own particular host range. While some phages can infect many species, or even those from different genera [21], most phages can only infect a very limited range of bacterial strains within a given species. This narrow specificity is likely an intrinsic characteristic of phages and a result of the long evolution of phage–bacteria interactions in nature, perhaps helping to strengthen the relationship between the two parties [22,23]. Owing to this specificity, phages can act as precision antibacterial agents. However, specificity can pose a hurdle for phage therapy, as it is necessary to determine whether any given bacterial target is susceptible to a particular type of phage prior to treatment. To overcome this issue, a combination of phages with different host ranges, brought together in a single cocktail, is currently the most common approach for a wider host spectrum [11,24]. Although this strategy is promisingly exploited, it still remains difficult to obtain coverage for all the potential strains of the bacterial species and bystander phages remain, which do nothing to add to the antibacterial action. The assembly of phage cocktails may also require the optimization of phage proportions to improve performance. Moreover, using cocktails composed of a diverse group of phages can pose challenges for manufacturing and approval processes. Therefore, it would be preferable, if practicable, to create synthetic phages with broaden host ranges rather than simply combine multiple phages.

In order to switch, or broaden, the host spectra of phages, several studies have focused on the fact that host range is associated, in most cases, with adsorption, the very first step of the phage life cycle, which requires phage components, such as tail fibers or other structural proteins, to bind to the receptors of target cells. The most prominent examples have concerned the expansion of the host range of the filamentous phage fd that normally infects *Escherichia coli* harboring F plasmid as the phage receptor. Marzari et al. [25] exploited the receptor-binding domain of the enterophage IKe gene *3* protein (g3p) that normally binds to the N or I pili. The domain was grafted to the N-terminus of the orthologous g3p of the phage fd. The resulting chimeric phage was able to infect *E. coli* harboring either F pilus or N pilus [25]. Another chimeric phage fd harboring the g3p fused with the orthologous protein (pIII^CTX^) of vibriophage CTX could infect *Vibrio cholerae* as well as *E. coli* [26].

Host range expansion has also been achieved in tailed phages that have more complex structures and larger genomes than filamentous phages. Lin et al. [27] have identified a recombinant phage (T3/7), a T3-based hybrid phage whose tail fiber gene *17* is a recombinant between those of T3 and T7. The hybrid T3/7 phage displayed better adsorption efficiency and a broader host range than both T3 and T7 phages [27]. This provided an insight into engineering tail fiber genes in the aforementioned *Caudovirales* phages, which led to the systematic swapping of the tail fiber genes using a yeast-recombineering toolkit [28]. The latter research group also demonstrated that an engineered *E. coli* phage T3 with a *Yersinia* tail fiber was able to unexpectedly infect both *E. coli* and *Yersinia pseudotuberculosis*. This was in contrast with an engineered *E. coli* phage T7 with a *Klebsiella* tail fiber that was only able to infect *Klebsiella.* This T7-based engineered phage is an example of a shifted or redirected host range based on common viral scaffolds. The intentional redirection of host specificity was first reported in 2005 [29]. Homologous recombination between a genome of the coliphage T2 and a plasmid encompassing the region around the tail fiber genes (*37* and *38*) of the similar coliphage PP01 resulted in the recombinant phage (T2ppD1) that possessed the tail fiber of PP01. T2ppD1 could not infect *E. coli* K12, but formed plaques on *E. coli* O157:H7 cells, demonstrating the desired change of host ranges. Later, the host range of T2 was expanded via the exploitation of long distal fiber genes from IP008 [30]. While this result provides a valuable platform for the expansion of the current phage reservoir, it is more useful for enhancing bacterial efficacy, in that well-characterized phage scaffolds can be harnessed to kill off-target bacteria by swapping the tail components of less-characterized phages that can infect the bacteria.

Thus, phage engineering based on the same, or similar, scaffolds can simplify the whole engineering process for the discovery and development of novel synthetic phages with broadened (and desired) host ranges and reduce the regulatory burden for human trials. Furthermore, functional phage particles can be newly synthesized from their genomes rather than traditionally obtained from pre-existing phage particles. Due to advances in synthetic biology toolkits and genome sequence information becoming available for a wider range of phages from numerous environmental habitats, this can readily be performed using genome recombineering and genetic manipulation techniques.

### 2.2. Phage Engineering to Minimize Host Immune Responses

Besides the narrow host spectra, another major limitation of phage therapy is its impact on the immune system. Phages can be recognized as immunogens by the host’s immune system, resulting in the inactivation of phages [18]. In addition, the materials released from bacterial lysis by lytic phages can trigger inflammatory responses in human hosts [31]. In light of concerns regarding these issues, two approaches need mentioning; the first is to reduce the immunogenicity of phages per se and the other is to reduce the collateral immunogenicity of bacterial elicitors derived from lysis by phages.

Merril et al. [32] designed a serial passage technique to select phage variants (mutants), which may avoid entrapment by the reticuloendothelial system and are thus able to remain in a host’s circulatory system for a longer period. By way of 10 serial injections of phages into mice, long-circulating phage λ mutants were isolated. These isolated mutants had a more enhanced antibacterial efficacy than the wild-type phage in the mouse models. This strategy required multiple endeavors and the mechanisms involved in reduced immunogenicity need further elucidation, in that a number of factors were likely responsible for the altered immunological and/or pharmacokinetic properties of the mutant phages. The λ mutants possessed a common substitution mutation from lysine to glutamic acid at the 158th residue (K158E) of the major phage head protein E [32]. The same group also demonstrated that the single K158E mutation was sufficient to exhibit the “long-circulating phenotype” [33]. A deeper understanding of phage epitopes and components in pharmacokinetics and pharmacodynamics (PK/PD) would better facilitate this type of engineering in the near future.

While the aforementioned strategy may immaculately help to avoid immune responses, there is still the problem of the release of toxic bacterial components during the infection cycle to overcome. This concern is of some importance, and was investigated by Matsuda et al. [34] via lysis-deficient phage therapy, using the phage T4-derived mutant (LyD), resulting in a decrease in the release of endotoxins as well as pro-inflammatory cytokines, such as TNF-α and IL-6. Mice treated with LyD exhibited attenuated pathophysiology, reduced bacteremia, and improved survival in a murine peritonitis-sepsis model for *E. coli* [34]. The recombinant *Staphylococcus aureus* temperate phage, P945, with its endolysin gene insertionally inactivated, displayed lysis-deficiency, but comparable bactericidal activity in vitro. This phage was fully able to rescue neutropenic mice from lethal infection by methicillin-resistant *S. aureus* (MRSA) [35].

In spite of limited knowledge about, or availability of, filamentous inophages, they seem to be an ideal workhorse for synthetic phage engineering, in that these phages reproduce not by lysis, but by secretion, without killing the host bacterium. Hagens and Bläsi [36] engineered the inophage M13 into M13R and M13S105 by incorporating the gene for the *Bgl*II restriction enzyme and the λ *S105* holin gene, respectively, to enhance the antibacterial activity of the filamentous phage. The engineered phage M13R produced a restriction enzyme, which caused nonrepairable double strand breaks in the bacterial chromosome, whereas the engineered phage M13S105 generated non-specific membrane lesions. The killing efficiency of both engineered phages was comparable to that of the lytic variant of the phage λ (λcI^−^). Nevertheless, endotoxin release by both phages was residual (about 6–7-fold) compared to that by M13, even though that by λcI^−^-infected cells increased (27-fold) at 4 h post-infection [36].

### 2.3. Phage Engineering to Maximize Antibacterial Efficacy

As shown by the case of M13R [36], and based on advances in tools and information, synthetic phage engineering has already been geared towards the enhancement of antibacterial efficacy, either directly through phages in their own right, or via improvements in the current antibiotic spectrum. This latter development, where phages are used as adjuvants to supplement or augment the bioactivity of the currently available antibiotics when used in tandem [37], also shows a great deal of promise.

Since the overexpression of LexA is known to suppress the SOS response, which supports DNA damage-inducible repair and the desultory emergence of antibiotic resistance [38], Lu and Collins [37] created an engineered phage (M13mp28) that overexpressed LexA3, a cleavage-resistant variant of the LexA repressor [39]. It was demonstrated that M13mp23 enhanced the antibacterial efficacy of bactericidal antibiotics such as ofloxacin, gentamicin, and ampicillin in vitro and in murine infections. In addition to the enhancement of antibacterial efficacy against vegetative cells in an ordinary culture, M13mp23 also exhibited an enhanced killing efficacy against antibiotic-resistant cells, persisters, and cells inside of structured biofilms [37]. This work is noteworthy in that it exploited the inophage, M13, as the workhorse for this purpose. This non-lytic, filamentous phage has a very small genome, whose replicative form (RF) in bacterial cells is just 6,407 base pairs. The RF of M13 is genetically tractable, acting like an ordinary plasmid in *E. coli*, allowing it to have multiple insertion sites that can accommodate different DNA fragments into the phage genome [40]. The phagemid systems are the plasmid systems containing M13 or f1 replication origins, which can be used for genetic engineering from which functional phages can be retrieved in the presence of helper phages such as R408 [41]. These features render inophages as the ideal synthetic phage engineering platform for the rapid translation of scientific knowledge into antibiotic adjuvants.

Basic knowledge of antibiotic resistance mechanisms can be simply translated to circumvent, or restore, the antibiotic-resistant bacteria using M13 as the synthetic biological chassis, based on the aforementioned features. As a matter of fact, one of the four archetype antibiotic resistance mechanisms, known as “target modification”, is the easiest way by which bacteria can acquire antibiotic resistance, simply due to point mutations on the target molecules. For example, resistance to rifampicin can be routinely generated in a laboratory, mostly via mutation(s) of its target molecule, the β subunit of the RNA polymerase core enzyme encoded by the *rpoB* gene [42]. Edgar et al. [43] engineered phage λ to reverse this bacterial drug resistance, thereby restoring sensitivity to antibiotics, with the same concept described as far back as 1951 for streptomycin [43,44]. The engineered phage delivered the wild-type (i.e., drug-sensitizing) *rpsL* and *gyrA* genes, which conferred sensitivity in a dominant fashion to streptomycin and nalidixic acid, respectively. Upon lysogenization by the engineered phage, the antibacterial susceptibilities of the resistant *E. coli* cells were restored and the minimum inhibitory concentration (MIC) values decreased 8-fold for streptomycin and 2-fold for nalidixic acid.

There are other straightforward, or more direct, strategies which can be used to compromise bacterial capability for growth and/or survival. These involve the insertion into phages of heterologous genes that are known to inhibit the bacterial growth and survival mechanisms, including stress responses and differentiation. One example is an engineered M13 equipped with toxin genes (*gef* and *chpBK*) that are presumed to be involved in altruistic cell death during nutrition deficiency [45,46,47,48]. Both the bacteria expressing Gef and those expressing ChpBK from the engineered M13 infection exhibited a reduction in colony forming units (CFUs) by 948- and 1579-fold, respectively. These phages displayed significant antibacterial efficacy in murine intraperitoneal infection by *E. coli* [47].

Approaches seeking to reduce virulence, termed antipathogenic or antivirulence approaches, have attracted growing attention for their potential to minimize the emergence of antibiotic resistance. These methods focus on disarming pathogenic bacteria along the key pathways of virulence or survival, are completely unlike traditional antibiotic mechanisms that target the key growth pathway, and, more importantly, drive resistance evolution and dissemination from the current “resistome” [49]. Since antipathogenics are, as either stand-alone or supplementary medications, intended to treat bacterial infections in a pathogen-specific manner, phages can be engineered to possess antipathogenic functions. Proof-of-concepts for antipathogenic drugs have been successfully extrapolated for chemical drug developments based on an in-depth understanding of cell-to-cell communications (quorum-sensing (QS)) in bacterial communities (biofilms) in infection settings. Lu and Collins [50] engineered the phage T7 to overexpress a biofilm degrading enzyme (DpsB), which was identified from *Actinobacillus actinomycetemcomitans*. Other biofilm-degrading enzymes that hydrolyze proteins or carbohydrates in the biofilm structure have also been harnessed for biofilm destruction [51]. The latter approach is also noteworthy in that it is effective against a wide range of bacterial strains, because the depolymerases are highly specific to the cognate host-derived polysaccharides and thus have limited effects on the natural biofilms of mixed bacterial species. Such engineered phages may be useful as anti-biofilm and anti-biofouling agents in industrial as well as clinical settings.

Unlike anti-biofilm activity that requires highly species- or strain-specific depolymerase enzymes, QS circuits may be the most promising antipathogenic target due to their critical roles in the formation of biofilm, virulence orchestration, and, more practically, the fact that they involve extracellular chemicals called autoinducers or quormones to monitor cell density in the population, which can be quenched or inactivated in the extracellular milieu [52]. Pei and Lamas-Samanamud [53] exploited an *aiiA* gene encoding the *Bacillus anthracis* lactonase that degrades a broad range of *N*-acyl homoserine lactones. The engineered phage (T7aiiA), expressing the *aiiA* gene transcribed from the Ф10 promoter, successfully compromised the QS of *P. aeruginosa* in a mixed biofilm with *E. coli*, which was included as the phage incubator [53].

That efforts have begun to change and broaden the strategies for deploying synthetic phage engineering is remarkable, in that all of the above approaches employ the phages as vehicles to deliver the effector genes rather than to use the phages as antibacterial agents. Although the major antibacterial functions stem from the installed effector genes, the phages are “intelligent” vehicles, since they are still capable of self-replication to amplify the activity of the delivered effectors. These strategies can be further strengthened by recent advancements in genome editing tools preponderantly based on the CRISPR-Cas system. The concept, from Hagens and Bläsi [36], of using a restriction enzyme to nonspecifically target a host genome, can be systemically expanded to “CRISPR antibacterials” that target a specific gene necessary for bacterial growth and survival and, more importantly, antibiotic resistance in any bacterial species with their available phages [36]. Owing to the precision cleavage of the CRISPR-Cas system, CRISPR-loaded phages will be able to cleave any target gene specifically, as designed. Citorik et al. [54] first made filamentous phage-based phagemid constructs of RNA-guided nucleases (RGNs) targeting one of the β-lactamase genes (*bla*_NDM-1_ and *bla*_SHV-18_), which encodes extended-spectrum and pan-resistance to β-lactams, respectively. When *E. coli* EMG2 carrying *bla*_NDM-1_ or *bla*_SHV-18_ were treated by phage particles (ΦRGN) packaged with one of the corresponding RGN phagemids, the viable cell counts decreased by 2 to 3 logs, while cells without the resistance genes were not affected at all. Similarly, quinolone-resistant *E. coli* harboring the *gyrA* mutation (D87G) were specifically affected by ΦRGN with the cognate RGN phagemid. The authors also verified the antibacterial efficacy of ΦRGN targeting a major virulence gene (*eae*) of the enterohemorrhagic *E. coli* O157:H7 (EHEC) in vivo using the *Galleria mellonella* infection model [54].

Bikard et al. [55] also designed a Cas9-based phagemid targeting the *aph-3* kanamycin resistance gene in a Gram-positive bacterium, *Staphylococcus aureus*. The modified *S. aureus* phage delivering the phagemid led to a 4-log CFU reduction in kanamycin-resistant bacteria, but did not elicit any effects on the kanamycin-sensitive bacteria. This target-specific antibacterial effect was also confirmed using the mouse skin colonization model [55].

The latter study illustrates another advantage of CRISPR antibacterials, showing that it is possible to engineer the phages with two (or more) crRNA guides to enable them to simultaneously target multiple chromosomal and/or episomal sequences. This strategy makes it possible to significantly mitigate the emergence of resistant clones that might evade the nuclease attack. This “multiplex targeting” has also been verified by additionally targeting chromosomal genes such as the *mecA* gene (for methicillin resistance) and the *sek* gene (superantigen enterotoxin) in the MRSA strain. It was also shown that the conjugative plasmids for antibiotic resistance in MRSA could be specifically eradicated by CRISPR targeting, suggesting that such engineered phages can balance the effects of antibiotic abuse by diminishing the chances of mobilome spread in natural habitats. CRISPIR antibacterials that have the ability to dramatically reduce plasmid content in bacterial populations without killing the bacteria could be used in a similar way to vaccines; to enhance herd immunity against plasmids that can transfer virulence and/or antibiotic resistance.

## 3. Phage Protein Engineering

Although many sagacious techniques have been developed to improve synthetic phage engineering, there is still much to be done in terms of refining drug development pipelines. Most importantly, the major disadvantages of phage particles, i.e., having narrow antibacterial specificities, the invocation of host immunity, and the mediation of horizontal gene transfer, have not been satisfactorily disentangled. In this regard, based on an increasing knowledge on the genomics and the life cycles of phages, phage components (in most cases, proteins), rather than the phages themselves, can be exploited as bioantibacterial agents. As seen in eukaryotic viruses, phage proteins are largely divided into two classes: Structural proteins (such as tails and capsids) and non-structural proteins (such as polymerases and specialized enzymes). The former may be represented by receptor switching in phage tail-like antibacterials (pyocins), while the latter are well exemplified by “phage lysins” or “enzybiotics” to engineer phage-derived non-structural enzymes (endolysins).

### 3.1. Lysin Engineering

In the process of developing new antibacterials and in light of the issues presenting for conventional phage therapies, phage enzyme engineering (herein collectively termed “enzybiotic engineering”) has increasingly attracted interest for the development of first-in-class antimicrobial biologics.

Nelson et al. [56] first coined the term “enzybiotics” for phage endolysins, describing their enzymatic and antibacterial properties toward group A streptococcal infections [57]. Among the various phage proteins, endolysins and, to a lesser extent, ectolysins are the most extensively studied enzymes for lysin engineering. Both enzymes are phage-derived peptidoglycan (PG) hydrolases that degrade the bacterial PG and thus have potent antibacterial activity [58]. While ectolysins are phage structural hydrolases that facilitate genome entry into bacterial cells during the early stages of the phage life cycle, endolysins are non-structural hydrolases that are essential for the release of phages during the final stages of the phage life cycle [59]. In this regard, almost all lytic phages possess their own endolysins, whereas ectolysins are found in some specialized muralytic phages and have been shown to display antibacterial activity [60,61,62,63]. Paul et al. [64] described an example of ectolysin engineering for the generation of a chimeric antibacterial enzyme (P128) by recombination of the 16-kDa C-terminal domain of the putative ectolysin (Orf56) of the *Staphylococcus* phage K with the C-terminal cell-wall targeting domain (SH3b) of the lysostaphin that binds to the polyglycine bridge in the PG crosslinks of some *Staphylococcus* species. In contrast to the large size of Orf56 (91-kDa), P128 exhibited a reduced size (27-kDa) and demonstrated a redirected antibacterial spectrum, killing the MRSA strain USA300 both in vitro and in vivo [64].

Due to the relative scarcity of ectolysins, phage endolysins have been predominantly utilized for lysin engineering. Unlike ectolysins, endolysins are essentially hydrolases working together with holins during the phage life cycle. It is well known, however, that endolysins are also capable of killing bacteria upon exogenous application as recombinant proteins. In addition, their narrower spectra (than those of traditional chemical antibiotics) can be redirected based on the diversity of the modular structures of endolysins from phage genomes (see below). This is an important advantage of endolysins, enabling them to overcome the limitations of the near-species specificity of endolysins, as well as allowing them to selectively kill the target bacteria, which helps to reduce the risk of resistance evolution often associated with the use of non-selective conventional antibiotics.

Historically, exogenous applications of endolysins and ectolysins have focused upon Gram-positive bacteria which lack an outer membrane (OM) barrier [65,66]. However, several natural and artificial peptides and chemicals, such as polymyxins with OM-permeabilizing properties, can be used in combination with endolysins. For example, LysABP-05 from an *Acinetobacter baumannii* phage exhibited antibacterial activity, which was significantly enhanced by the presence of an OM-permeabilizing peptide, colistin [67]. For better endolysin-derived biologic antibacterials, however, Briers et al. [68] described the proof-of-concept that an OM-permeabilizing peptide can be fused to an endolysin in a single polypeptide to target Gram-negative bacteria [68]. Hence, “Artilysins”, phage-derived endolysins engineered to display OM-penetrating functions, were developed. Artilysins could be further optimized to show high and rapid bactericidal activity against *P. aeruginosa* and *A. baumanii* [69], suggesting that Artilysins could be another engineering platform for better antibacterials.

**Figure 2:**
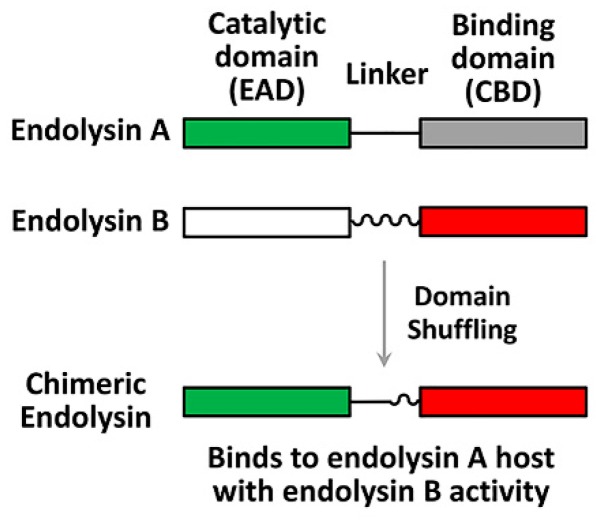
Endolysin engineering strategy. The modular structures of endolysins are shown; catalytic domains (enzymatically active domain (EAD)) of endolysin A and endolysin B (green and white rectangles) are originally connected to their binding domains (cell wall-binding domain (CDB)) (grey and red rectangles) via cognate linkers (straight and wavy lines). An engineered chimeric endolysin can be created by domain shuffling using the endolysin A EAD and the endolysin B CBD with an appropriate linker.

Endolysins display a characteristic modular structure, consisting of at least two functional domains with a linker in-between (Figure 2): The N-terminal enzymatically active domain (EAD) and the C-terminal cell wall-binding domain (CBD). CBDs define the specificity, whereas EADs define the murlaytic (i.e., antibacterial) activity. The modular structure of endolysins provides a desirable platform for lysin engineering for better antibacterial biologics, which can be done to: (1) Redirect or broaden the specificity, (2) improve the catalytic efficiency, and/or (3) enhance the in vivo (serum) stability. For example, Díez-Martínez et al. [70] designed four chimeric endolysins (Cpl-117, 177, 711, and 771) by shuffling the domains (EAD, linker, and CBD) from the pneumococcal phage lysin (Cpl-1) and an engineered lysin (Cpl-7S). Among these four chimeric endolysins, Cpl-711, possessing the EAD from Cpl-7S and the linker and the CBD from Cpl-1, displayed the most potent lytic activity [70,71]. Recently, Seijsing et al. [72] reported that the stability of endolysin was extended via fusion with the albumin binding domain (ABD), without affecting antibacterial activity. These examples demonstrate the feasibility of new and more potent endolysins by shuffling the domains of the preexisting endolysins, or by fusion with the functional domains of other proteins.

### 3.2. Pyocin Engineering

**Figure 3:**
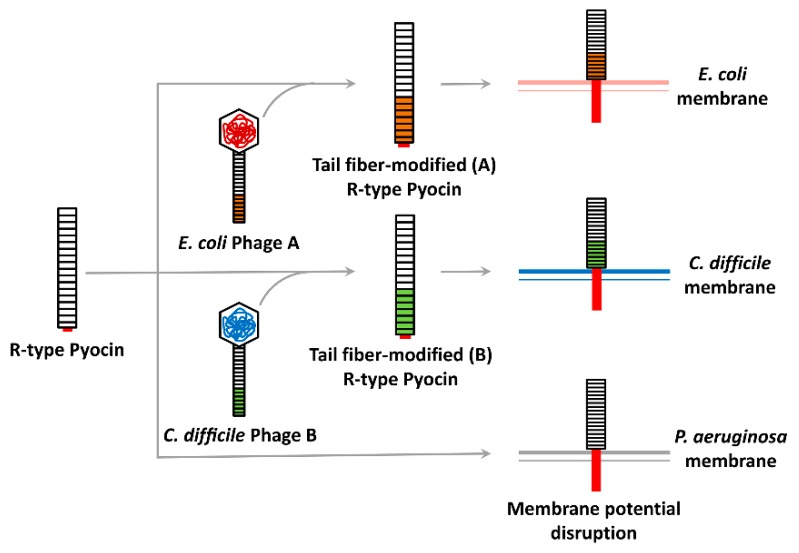
Pyocin engineering strategy and mode of action. The target of an R-type pyocin can be shifted from *Pseudomonas aeruginosa* to *Escherichia coli* or *Clostridium difficile* by tail shuffling. The tail fibers of either (**A**) *E. coli* phage or (**B**) *C. difficile* phage are fused to the R-pyocin, resulting in modified R-pyocins that can bind to respective target bacterial membranes. Once the modified R-pyocins reach each hosts’ membranes, sheath contraction induces core tube penetration and disrupts the membrane potential (red bars).

R (rod or rigid)-type pyocins, R-tailocins, or R-pyocins, although not directly produced by phages, have been considered to be an attractive option for bioantibacterial development, owing to their fast and highly efficient bactericidal activity that disrupts bacterial membrane potential [73,74]. R-pyocins are bacteriocins found in almost all *P. aeruginosa* isolates and may direct the competitive growth advantages of *P. aeruginosa* strains in mixed populations [75,76]. They are evolutionarily derived from phages and thus are composed of long tube cores covered with sheaths that are connected to a baseplate, to which receptor binding proteins (RBPs) are attached. This structure is quite similar to that of the tail of phage λ, and the type IV secretion system [77]. R-pyocins are known to bind to the core polysaccharide of the OM in *P. aeruginosa* via RBPs [74], which induces the contraction of the sheath and initiates the penetration of the core tube into the cells [77,78,79]. As a result, pores are created to disrupt membrane potential, which results in rapid cell death [79]. R-pyocins were proven to cure *P. aeruginosa* infections in mouse peritonitis models [80] and many studies have focused on pyocin engineering to redirect the antibacterial spectrum of R-pyocins. A study by Williams et al. [81] first described pyocin engineering to shift the target from *P. aeruginosa* to *E. coli* simply though the substitution of the tail fiber RBP, as shown in Figure 3. Chimeric pyocins were created by fusion of the N-terminus of the R-pyocin tail fiber and the C-terminus of the P2 phage tail fiber. As a result, the chimeric pyocin killed *E. coli*, while it lost its killing activity against *P. aeruginosa* [81]. Later, the same rationale was successfully applied to kill Shiga toxin-producing *E. coli* (STECs) such as EHEC, O157:H7 and a highly virulent enteroaggregative non-O157 STEC, *E. coli* O104:H4 [82,83]. Unlike traditional antibacterials, the engineered pyocins did not cause a release of Shiga toxin from the EHEC in a rabbit infection model, reporting a great reduction in disease symptoms [84]. Recently, R-pyocin engineering has also been applied to control *Clostridium difficile* [85]. The well-characterized R-pyocin (i.e., diffocin), CD4 was engineered to kill other *C. difficile* strains; the original RBP was replaced with a new RBP discovered from a prophage of the target strains by genome mining. Furthermore, due to its stability in vivo, R-pyocin engineering has great potential, especially in oral administration, to target intestinal pathogens, or to remodel the intestinal microbiome [79].

R-pyocin engineering is similar to lysin engineering, in that both R-pyocins and endolysins have modular structures that can be shuffled or evolved based largely on the available knowledge regarding them and the high diversity of the phages and bacterial genomes. Nevertheless, R-pyocins may have more disadvantages than engineered endolysins due to their large size and ordered structure. This is because R-pyocins are as large as phages and thus may have more epitopes that invoke host immunity than lysins do and R-pyocins are highly structured nanotubes which may thus have little sequence space for directed evolution and further engineering. Despite these disadvantages, there is still ample opportunity for engineering novel R-pyocins in possession of unique niches that are distinct from both phages and lysins, which would definitely help reduce the increasing incidence of antibiotic resistance obstructing therapeutic efforts around the world.

## 4. Phage-Inspired Antibacterial Design

**Figure 4:**
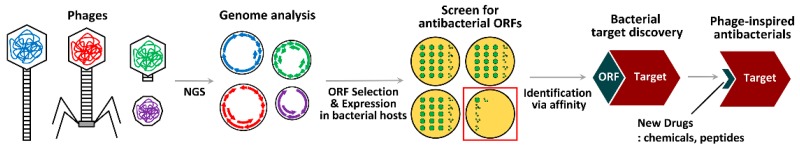
Work flow of phage-inspired antibacterial design. Once phages with strong antibacterial activity are identified, their whole genomes are sequenced and analyzed for open reading frame (ORF) selection. The selected ORFs are expressed in target bacterial hosts to screen for antibacterial activity based on growth or virulence inhibition. Then, the gene product of an ORF hit (dark green) is exploited to fish out the host target based on protein–protein interactions. The discovered host target, the phage-inspired bacterial target, serves as a molecular scaffold for new drug screening. Finally, chemicals or peptides that bind to the host target can be isolated and further developed as phage-inspired antibacterials.

There exists another approach which utilizes phages and phage materials, not with existing antibacterials as discussed above, but involving the search for new antibacterials. This method uses an examination of the life cycles of various phages and their respective identifiable proteins to garner inspiration for new concepts. Phages are rapidly evolving biological systems that can adapt themselves to circumvent any changes in the host’s bacterial physiology by directing, or hijacking, host mechanisms for their own replication. Phages possess highly sophisticated means to modulate and/or compromise bacterial physiology, which involve the workings of the phage protein(s) to target the most vulnerable bacterial proteins in the central pathways for growth and/or survival. This is the rationale behind the concept of “phage-inspired antibacterial design”, which can be initiated as a result of the elucidation of bacterial targets that have previously been known only to the phages themselves and unbeknownst to us (Figure 4).

The first proof-of-concept study in this area was conducted by Liu et al., utilizing phage genomics [86]. By way of the sequencing of 26 *Staphylococcus aureus* phages, 31 proteins that could inhibit bacterial growth by functional screen upon overexpression in bacterial cells were identified. Among them, the ORF104 of phage 77 (77ORF104) was selected to identify its bacterial target through protein–protein interaction. 77ORF104 specifically interacted with DnaI, an essential bacterial protein, during the initiation of DNA replication. The most momentous result in this study was the discovery, when using 125,000 compounds, of a chemical hit that impaired the interaction between 77ORF104 and DnaI from the high-throughput screen. This hit could inhibit the growth of bacteria via the same mechanism as 77ORF104. The research team hypothesized a phage-inspired antibacterial design with a new antibacterial mechanism from the antibacterial hit, which could be done by elucidating the new antibacterial target (i.e., DnaI) that followed the identification of the phage protein (i.e., 77ORF104).

This concept can be significantly expanded considering the abundance of fully sequenced phage genomes in the current databases that contain many predicted open reading frames (ORFs) that show little or no homology to proteins in the extant sequence databases. There are several studies that have uncovered phage proteins that directly bind to the host proteins required for the core cellular processes in bacterial growth and virulence, briefly summarized in Table 1, as recently reviewed by de Smet et al. [87]. All of these studies have identified the antibacterial phage proteins and their target proteins or the submolecular regions of the target proteins, which account for the antibacterial mechanisms. These suggest, as inspired from the phage life cycles, the previously unknown antibacterial targets for new antibacterial development.

Our research group has identified one such protein from the temperate siphophage, D3112, that infects *P. aeruginosa* strains [99]. The phage-encoded protein (gp05 or Tip) specifically inhibits the polar localization of the bacterial ATPase (PilB) involved in type IV pilus (TFP) assembly. TFPs are required for surface adherence, twitching motility and biofilm formation and thus also for full virulence. The antibacterial efficacy of D3112 has been demonstrated using *Drosophila* and mouse infection models [101], suggesting that targeting TFPs is an effective antipathogenic maneuver, since TFPs are required for virulence but not for the growth of *P. aeruginosa*. Since ATPases are relatively conserved in motility and secretion mechanisms crucial for bacterial pathogenesis, the phage encoded Tip protein could be translated into new antibacterials either with its anti-PilB mechanism to identify chemical hits as in Liu et al. [86], or via its functional modality being used to inspire new molecular scaffolds for peptide hits.

The abundance of structural and molecular biological characterizations of the antibacterial mechanisms of phage proteins and their interactions with their bacterial partners would translate into first-in-class antibacterial drugs. Although it is unavoidable that these drugs would eventually provoke antibacterial resistance, they clearly differ from conventional antibiotics whose bacterial targets (such as PG synthesis, protein synthesis, and nucleic acid synthesis) are shared with the preexisting natural compounds or the xenobiotic derivatives. This difference is important, in that the new drugs, unless they share targets with traditional antibiotics, would possess a unique mechanism of action learned from phage life cycles and not be affected by the current reservoir of the antibiotic resistome that might act as a “phantom menace” [102] for newly discovered antibiotics. Therefore, the concept of phage-inspired antibacterial design could greatly facilitate the discovery of new classes of antibacterial chemicals or biologics to fulfill the requirements necessary for antibacterial discovery and development in the 21st century.

## 5. Conclusions

Phages are the most predominant biological entities on the planet. The recent explosion of sequencing information concerning them, resulting from the developing field of NGS and improved electron microscopy techniques, reveal that they possess a prodigious diversity. In essence, phages have long been evolving and adapting themselves to optimize their life cycles within their hosts’ bacterial physiology. This has inspired the conceptual framework of phage-derived antibacterials, which can be brought to fruition with new synthetic biology techniques as well as the maturation of phage biology itself as a discipline. Being the simplest biological system, phages are the ideal bioplatform for fast and precise engineering. Actually, phages have already been widely exploited for non-medical purposes that include anti-septic agents, diagnostic tools, and delivery systems [103]. However, they need to be carefully addressed for medical purposes due to the potential risk as “live” entities and ethical pitfalls, especially for engineered phages [9]. Nevertheless, the rapid emergence of drug-resistant bacteria has jeopardized the efficacy of chemical antibiotics and consequently led us to the precipice of the greatest and most urgent global health crisis—that posed by antibacterial resistance. This crisis, together with technological advancements in phage engineering, has enkindled the development of phage research and engineering for medical purposes.

Although there have been waves of advancements in phage research and engineering, in this review, we have briefly highlighted three types of study based on how the components of phages are subjected to exploitation. The first type is where the phages themselves are exploited by synthetic phage engineering. In the second category, the protein components of phages are utilized directly for phage protein engineering. In the last grouping of studies, the protein components of the phages are utilized indirectly to inspire new antibacterial designs. Despite the clear differences between these methods, and the various strengths and weaknesses which each method possesses, what all of these approaches have in common is the need for further optimization so as to overcome practical issues facing phage-derived biologics, while leaving the essence of the advantages of phages as antibacterials largely untouched. Most importantly, when compared to chemical antibiotics, phage-derived antibacterials may possess much room for design and redesign, allowing for continuous improvements in biologics (i.e., biobetters). Phages may also be subjected to an array of directed evolution and/or conjugation with other modalities (such as antibodies or ABDs) to enhance activity, safety, and stability. This is particularly the case when a comparison is made with vaccines and recombinant protein biologics markets, because phage biology is still in its infancy and phage types are incredibly diverse in nature, all awaiting our extensive investigations. More importantly, most of the phage engineering to date has only involved a few genetically tractable phage species, such as M13 and T7, infecting certain strains of the model bacterium, *E. coli*. It is most likely that the tremendous abundance of phages needs to be explored not only with model bacteria, but also with drug-resistant bacteria such as ESKAPE pathogens, including *P. aeruginosa* and *S. aureus* [104,105], which are more important for medical purposes. The enlargement of NGS-based genome information, the enhancement of random mutagenesis-based sequence spaces, and the improvement of molecular microbiology-based engineering tools for these bacterial systems, would all accelerate the synthetic biology and engineering cycles (i.e., design-build-test-learn) for newly created, tailor-made phage antibacterials.

Finally, we now need to pay more attention to innovatively recruit all phage resources for phage applications. For example, although they have been underappreciated compared to better-known lytic DNA phages, we should pay attention to temperate DNA phages, and even RNA phages, for bioplatforms. Given that temperate phages do not rapidly kill the host bacteria, instead actively modifying the properties and behaviors of them, they are likely armed with genetic resources which have evolved to actively manipulate the host’s bacterial physiology and may provide clues concerning new antibacterial targets and proteins. As exemplified in our previous study, we identified a phage protein (Tip) and its new antipathogenic target (PilB), from an unmodified temperate phage that displayed therapeutic efficacy in acute infections caused by *P. aeruginosa* [99,101]. In that temperate phages’ life cycles have focused on establishing a co-flourishing symbiosis with that of their host bacteria, they may be advantageous over lytic phages for reducing the emergence of antibacterial resistance. Likewise, RNA phages can also be utilized once their life cycles and gene functions are more extensively investigated, mainly because they do not transfer bacterial genetic elements to drive the host’s evolution. They have RNA genomes and their capsid proteins are known to specifically interact with these genomes for phage assembly, as in the leviphage, MS2 [106,107]. Considering the diversity of eukaryotic RNA viruses based on the higher mutation rates of RNA replicases and the current limitations of NGS-based metagenomics, which have concentrated on DNA rather than RNA, it is undeniable that RNA phages have not been fully explored. A recent study revealed that a carrier state (pseudolysogeny) of *P. aeruginosa* could be established upon infection by a leviphage (LeviOr01) [108], although the molecular mechanisms still remain elusive. It is also interesting that the propagation of another *P. aeruginosa* leviphage (PP7) clearly differed from other strains with the susceptible receptor [109]. Despite having the simplest genome organization, leviphages still need to be scrutinized for phage research and engineering, which could be facilitated by reverse genetic and epitranscriptomic analyses of phage life cycles.

In summary, owing to their simplicity, plasticity, and diversity, interest in phages has experienced a recent resurgence, and there now exists great potential utility for new-paradigm antibacterial discovery through synthetic biology tools. Nonetheless, for real-world applications there remain critical hurdles for engineered phage-derived bioantibacterials which need to be reconciled within the permitted industrial and regulatory frameworks for pharmaceutical production, licensing, and approval. Therefore, no doubt spurred on by a tremendous medical need worldwide, continued research in related disciplines is required to help address the remaining concerns still seen with phage-derived biologics and to realize their significant potential for human welfare.

## Figures and Tables

**Table 1:** Examples of vulnerable bacterial targets by phage proteins.

Phage Protein	Length and Size	Host Species	Host Target	Target Process	Reference
N4 Gp8	71 aa/8.1 kDa	*Escherichia coli*	HolA (DNA polymerase)	Replication	[88]
ΦX174 lysis protein	91 aa/10.6 kDa	*Escherichia coli*	MraY (PG precursor translocase)	Cell wall synthesis	[89]
T7 Gp0.4	51 aa/5.8 kDa	*Escherichia coli*	FtsZ (cell division protein)	Cell division	[90]
T7 Gp0.7 ^a^	359 aa/41.1 kDa	*Escherichia coli*	RpoC (RNA polymerase)	Transcription	[91]
T7 Gp2	64 aa/7.2 kDa	*Escherichia coli*	RpoD (RNA polymerase)	Transcription	[92]
T7 Gp5.5	99 aa/11.2 kDa	*Escherichia coli*	H-NS (histone-like protein)	Transcription	[93]
T7 Gp5.7	69 aa/7.4 kDa	*Escherichia coli*	RpoS (RNA polymerase)	Transcription	[94]
14-1 Gp12	310 aa/33.9 kDa	*Pseudomonas aeruginosa*	RpoA (RNA polymerase)	Transcription	[95]
LUZ19 Gp25.1 ^b^	116 aa/13.6 kDa	*Pseudomonas aeruginosa*	RpoC (RNA polymerase)	Transcription	[96]
LUZ24 Mip (Gp4)	46 aa/5.7 kDa	*Pseudomonas aeruginosa*	MvaT (H-NS-like protein)	Transcription	[97]
ΦKZ Dip (Gp37)	273 aa/30.9 kDa	*Pseudomonas aeruginosa*	Rne (RNase E degradosome)	RNA degradation	[98]
D3112 Tip (Gp5)	136 aa/14.4 kDa	*Pseudomonas aeruginosa*	PilB (TFP assembly ATPase)	Motility	[99]
77 ORF104	52 aa/6.2 kDa	*Staphylococcus aureus*	DnaI (DNA primosome)	Replication	[86]

^a^ T7 gp0.7 is a serine/threonine kinase that phosphorylates RpoC to aid the Gp2 function. T7 gp0.7 also affects multiple host proteins [100]; ^b^ LKA1 gp36 and LKD16 gp25b were also identified based on direct interactions with RpoC in the same study.
